# Identity and function of an essential nitrogen ligand of the nitrogenase cofactor biosynthesis protein NifB

**DOI:** 10.1038/s41467-020-15627-9

**Published:** 2020-04-09

**Authors:** Lee A. Rettberg, Jarett Wilcoxen, Andrew J. Jasniewski, Chi Chung Lee, Kazuki Tanifuji, Yilin Hu, R. David Britt, Markus W. Ribbe

**Affiliations:** 1 Department of Molecular Biology and Biochemistry, University of California, Irvine, CA 92697-3900 USA; 2 Department of Chemistry, University of California, Davis, CA 95695 USA; 3 Department of Chemistry, University of California, Irvine, CA 92697-2025 USA; 4 Present Address: Department of Chemistry and Biochemistry, University of Wisconsin, Milwaukee, WI 53211 USA

**Keywords:** Bioinorganic chemistry, Structural biology, Biosynthesis

## Abstract

NifB is a radical *S*-adenosyl-L-methionine (SAM) enzyme that is essential for nitrogenase cofactor assembly. Previously, a nitrogen ligand was shown to be involved in coupling a pair of [Fe_4_S_4_] clusters (designated K1 and K2) concomitant with carbide insertion into an [Fe_8_S_9_C] cofactor core (designated L) on NifB. However, the identity and function of this ligand remain elusive. Here, we use combined mutagenesis and pulse electron paramagnetic resonance analyses to establish histidine-43 of *Methanosarcina acetivorans* NifB (*Ma*NifB) as the nitrogen ligand for K1. Biochemical and continuous wave electron paramagnetic resonance data demonstrate the inability of *Ma*NifB to serve as a source for cofactor maturation upon substitution of histidine-43 with alanine; whereas x-ray absorption spectroscopy/extended x-ray fine structure experiments further suggest formation of an intermediate that lacks the cofactor core arrangement in this *Ma*NifB variant. These results point to dual functions of histidine-43 in structurally assisting the proper coupling between K1 and K2 and concurrently facilitating carbide formation via deprotonation of the initial carbon radical.

## Abstract (web summary)

NifB is a radical SAM enzyme involved in the biosynthesis of the Mo-nitrogenase cofactor, which is responsible for the ambient conversion of N_2_ to NH_3_. Here, the authors identify and uncover the function of a His43 residue as an essential nitrogen ligand of NifB in cofactor biosynthesis.

## Introduction

NifB plays a key role in the biosynthesis of the active site of Mo-nitrogenase, an [(*R*-homocitrate)-MoFe_8_S_9_C] metallocofactor (designated M-cluster) that catalyzes the ambient conversion of dinitrogen (N_2_) to ammonia (NH_3_)^1–6^. A member of the radical *S*-adenosyl-L-methionine (SAM) enzyme family^7,8^, NifB is a monomeric protein carrying three [Fe_4_S_4_] clusters:^9–12^ one, designated the SAM-cluster, is ligated by a canonical CxxxCxxC motif and coordinates the SAM-binding [Fe_4_S_4_] cluster; the other two, designated the K-cluster, are ligated by a number of conserved ligands and supply the full complement of iron (Fe) atoms for the biosynthesis of an 8Fe precursor to the mature cofactor. Previous studies of the NifB proteins from *Azotobacter vinelandii* and *Methanosarcina acetivorans* have led to the proposal that NifB utilizes a unique, radical SAM-dependent mechanism for carbide insertion concomitant with the transformation of the K-cluster to an [Fe_8_S_9_C] cluster (designated the L-cluster), which represents both an 8Fe precursor and an all-Fe core of the M-cluster^13–16^. This process begins with an S_N_2-type transfer of a methyl group from a SAM molecule to the K-cluster (Supplementary Fig. 1a, ➀), and it is followed by hydrogen abstraction of the methyl group by a 5′-deoxyadenosyl (5′-dA•) radical that is derived from the homolytic cleavage of a second SAM molecule (Supplementary Fig. 1a, ➁). The resultant methylene radical then undergoes further deprotonation/dehydrogenation to yield a μ_6_-coordinated interstitial carbide concomitant with the radical-chemistry-based coupling and rearrangement of the two [Fe_4_S_4_] modules of the K-cluster (designated K1- and K2-cluster, respectively) into an [Fe_8_S_9_C] L-cluster, which represents an all-Fe core of the M-cluster except for the substitution of one Fe atom for Mo/homocitrate at one end of the cluster (Supplementary Fig. 1a, ➂). Once generated, the L-cluster is passed from NifB onto NifEN, the next biosynthetic scaffold, where it is matured into a fully complemented M-cluster upon insertion of Mo and homocitrate by NifH prior to the delivery of M-cluster to its final binding site in NifDK, the catalytic component of Mo-nitrogenase (Supplementary Fig. 1a, ➃)^17–22^.

The unique radical chemistry that occurs on NifB during the nitrogenase cofactor core assembly makes this protein an attractive subject of investigation, as knowledge in this regard will shed light on the unusual synthetic route to a high-nuclearity metallocofactor that is biologically important and chemically elusive. Previously, we have identified three sets of Cys ligands—three Cys per set—for the three [Fe_4_S_4_] clusters in *M. acetivorans* NifB (designated *Ma*NifB), namely, the SAM- cluster (Cys^50^, Cys^54^, and Cys^57^), the K1-cluster (Cys^30^, Cys^63^, and Cys^129^) and the K2-cluster (Cys^264^, Cys^274^, and Cys^277^) (Supplementary Fig. 1b, c)^11^. Further, we have established the coexistence of SAM- and K2-clusters as a prerequisite for methyltransfer and hydrogen abstraction to occur and pinpointed the K2-cluster as the site for methyl attachment and the subsequent hydrogen abstraction from the methyl group by a 5′-dA• radical^11^. Perhaps even more excitingly, using pulse EPR techniques, we have identified a nitrogen atom from a His residue as the fourth ligand for the K1-cluster that is lost upon coupling between the K1- and K2-clusters into an L-cluster^11^. This observation suggests an important role of the His/nitrogen ligand in generating the nitrogenase cofactor core (i.e., the L-cluster), particularly given the ability of His to undergo protonation/deprotonation, which either provides a release mechanism for the L-cluster onto the next biosynthetic apparatus or allows this residue to participate in the further deprotonation/dehydrogenation of the carbon radical to give rise to a central carbide. As such, it is crucial to identify this nitrogen ligand and probe its function in the process of nitrogenase cofactor core formation.

Here we use a combination of site-directed mutagenesis and pulse EPR spectroscopy to show that the His^43^ residue is the specific nitrogen ligand for the K1-cluster of *Ma*NifB. Our biochemical and EPR analyses demonstrate the essential role of His^43^ in the formation of the nitrogenase cofactor core structure, although substitution of this residue with Ala does not impact early steps of carbide insertion leading to the initial hydrogen atom abstraction of the K2-associated methyl group. Our XAS/EXAFS analysis further reveals a shortened distance between the K1- and K2-clusters upon substitution of His^43^ with Ala, as well as a further processing of these clusters into an intermediate between the K- and L-clusters upon incubation with SAM. These observations point to a dual function of His^43^ in positioning/orienting the K1-cluster relative to the K2-cluster for proper coupling and deprotonating the initial carbon radical for carbide formation.

## Results

### The identity of the histidine ligand of the K1-cluster

Sequence analysis of *Ma*NifB revealed the presence of three highly conserved His residues, His^28^, His^43^ and His^219^, which could potentially serve as the nitrogen ligand for the K1-cluster on NifB^*Ma*^ (Supplementary Fig. 1b). On the basis of this analysis, three *Ma*NifB variants were heterologously expressed in *Escherichia coli*. Designated *Ma*NifB^H28A^, *Ma*NifB^H43A^ and *Ma*NifB^219A^, respectively, each variant has one of the three conserved His residues substituted with Ala. Purified *Ma*NifB variants, like their wildtype counterpart (designated *Ma*NifB^wt^), are monomers of ~38 kDa (Supplementary Fig. 2a). Moreover, upon FeS reconstitution, *Ma*NifB^H28A^, *Ma*NifB^H43A^, and *Ma*NifBH^219A^ have Fe contents comparable to that of *Ma*NifB^wt^, all of which carry three [Fe_4_S_4_] clusters (i.e., the SAM-, K1- and K2-clusters) per protein (Supplementary Fig. 2b). Thus, a loss of the His ligand does not seem to impact the ability of *Ma*NifB to ligate any of the three [Fe_4_S_4_] clusters, likely due to the 3-Cys-coordination of these clusters that is sufficient to secure them in place.

**Fig. 1:**
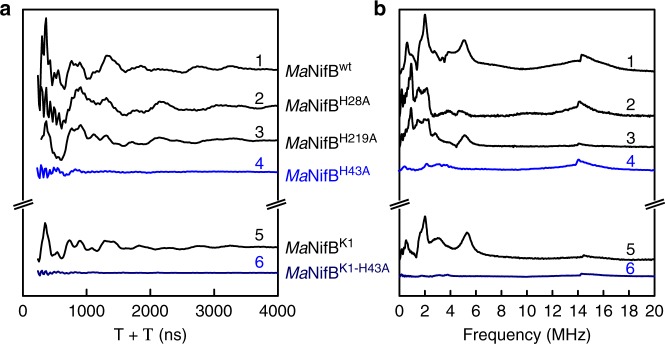
Three-pulse ESEEM spectra of dithionite-reduced *Ma*NifB proteins. **a** Time domain and (**b**) fast Fourier transformed (FFT) spectra of *Ma*NifB^wt^ (1), *Ma*NifB^H28A^ (2), *Ma*NifB^H219A^ (3), *Ma*NifB^H43A^ (4), *Ma*NifB^K1^ (5) and *Ma*NifB^K1-H43A^ (6). The time domain spectra of the His-ligand containing samples (i.e., 1, 2, 3, and 5) have modulations from ^14^N that appear as peaks in the fast Fourier transformed (FFT) spectra between 1 and 6 MHz. The sharp modulations between 250 and 500 ns in the time domain and the resulting broad peak near 14 MHz in the FFT are from nearby weakly coupled protons. The ESEEM spectra were recorded at 10 K, τ = 128–144 ns, π/2 = 12 ns, and 9.3366 GHz. The experiment was performed three times independently (*n* = 3 independent samples), and representative results are shown in the figure. All protein samples have a concentration of 15 mg mL^−1^. The CW EPR spectra of these dithionite-reduced *Ma*NifB proteins are shown in Supplementary Fig. 3.

To assess whether a nitrogen ligand is still present in these *Ma*NifB variants, pulse EPR spectroscopy—specifically, three-pulse electron spin echo envelope modulation (3P-ESEEM)—was used to observe modulations to the time domain spectrum and corresponding peaks in the fast Fourier transformed (FFT) spectrum that can be assigned to the coordinating nuclei (Fig. 1; also see Supplementary Fig. 3). Consistent with our previous observation^11^, *Ma*NifB^wt^ shows deep modulations in the time domain of the ESEEM spectrum (Fig. 1a, trace 1) and corresponding intensity between 0 and 6 MHz in the FFT (Fig. 1b, trace 1), which have been previously assigned to the hyperfine and quadrupole couplings of a cluster-ligated ^14^N nucleus^11^. Similar modulations and intensities are present in the ESEEM spectrum and FFT of *Ma*NifB^K1^, a variant carrying only the K1-cluster but no SAM- and K2-clusters (because of substitutions of the Cys ligands of the SAM- and K2-clusters with Ala^11^), affirming the previous assignment of the nitrogen ligand to the K1-cluster (Fig. 1a, b, trace 5). While similarly deep modulations and intensities are observed in the ESEEM spectra and FFTs of *Ma*NifB^H28A^ and *Ma*NifB^H219A^ (Fig. 1a, b, traces 2, 3), these features are clearly absent from the ESEEM spectrum and FFT of *Ma*NifB^H43A^ (Fig. 1a, b, trace 4), suggesting His^43^ as the nitrogen ligand for the K1-cluster. To seek further support for this assignment, *Ma*NifB^K1-H43A^—another *Ma*NifB variant carrying only the Cys ligands of K1 along with a substitution of His^43^ with Ala—was heterologously expressed in *E. coli*. The purified *Ma*NifB^K1-H43A^ demonstrates the same subunit composition as *Ma*NifB^K1^, as well as the same Fe content that is consistent with the presence of one [Fe_4_S_4_] cluster (i.e., the K1-cluster) per protein upon FeS reconstitution (Supplementary Fig. 2a, b). However, contrary to *Ma*NifB^K1^ (Fig. 1a, b, trace 5), *Ma*NifB^K1-H43A^ does not show deep modulations and intensities in its ESEEM spectrum and FFT (Fig. 1a, b, trace 6), firmly establishing His^43^ as the nitrogen ligand that specifically coordinates the K1-cluster.

### The role of histidine ligand in L-cluster maturation

**Fig. 2:**
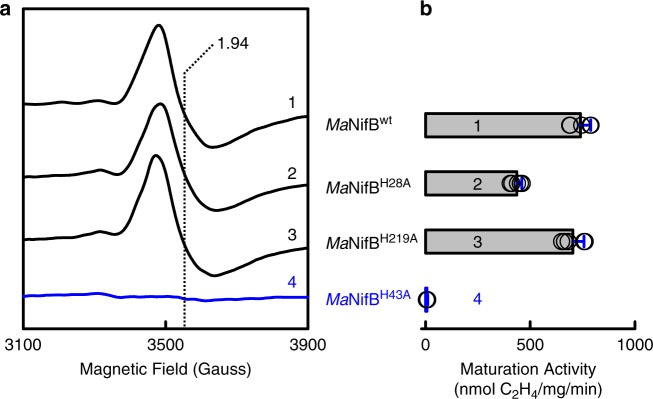
Conversion of L-cluster to M-cluster on *Ma*NifB proteins. **a** EPR spectra of IDS-oxidized *Ma*NifB^wt^ (1), *Ma*NifB^H28A^ (2), *Ma*NifB^H219A^ (3), and *Ma*NifB^H43A^ (4) upon addition of SAM. Formation of the L-cluster was monitored by the appearance of an L-cluster-specific *S* = 1/2 signal at *g* = 1.94 (dashed vertical line). All protein samples have a concentration of 15 mg mL^−1^. The EPR spectra were recorded at 5 mW and 20 K. **b** M-cluster maturation activity of *Ma*NifB^wt^ (1), *Ma*NifB^H28A^ (2), *Ma*NifB^H219A^ (3), and *Ma*NifB^H43A^ (4). The activity of M-cluster maturation was determined on the basis of the C_2_H_2_-reducing activity of reconstituted NifDK, using SAM-treated *Ma*NifB proteins as the M-cluster sources. The EPR analysis was performed three times independently (*n* = 3 independent samples), and representative results are shown in (**a**). The maturation assay was performed five times independently (*n* = 5 independent samples), and data are presented as mean ± S.D. (**b**). See Methods for the detailed composition of maturation assays.

Continuous wave (CW) EPR analysis provided the first insights into the function of His^43^ in the cofactor core assembly process. Consistent with the coupling and rearrangement of the K1- and K2-clusters into an L-cluster, an L-cluster-specific, *g* = 1.94 signal^9,10,15^ is observed in the spectra of *Ma*NifB^wt^, *Ma*NifB^H28A^, and *Ma*NifB^H219A^ upon incubation of these proteins with SAM (Fig. 2a, traces 1–3). In contrast, the *g* = 1.94 signal is absent from the spectrum of *Ma*NifB^H43A^ following the same treatment, suggesting a lack of K- to L-cluster transformation on this protein after incubation with SAM (Fig. 2a, trace 4). In support of this assignment, the SAM-treated *Ma*NifB^wt^, *Ma*NifB^H28A^, and *Ma*NifB^H219A^ can be used as M-cluster sources for the subsequent reconstitution and activation of apo-NifDK in an in vitro assay; whereas the SAM-treated *Ma*NifB^H43A^ cannot support the reconstitution and activation of apo-NifDK in the same assay (Fig. 2b). Interestingly, the activity of *Ma*NifB^H28A^ in this assay is ~41% less than those of *Ma*NifB^wt^ and *Ma*NifB^H219A^, suggesting a possible involvement of His^28^ in the K- to L-cluster conversion due to the extremely close location of this residue to one of the Cys ligands (Cys^30^) of the K1-cluster in the primary sequence (see Supplementary Fig. 1b) and, consequently, the tertiary structure of NifB. More importantly, the abolished activity of in *Ma*NifB^H43A^ in the K- to L-cluster transformation points to a critical role of His^43^ in coupling the K1- and K2-clusters into an 8Fe L-cluster.

**Fig. 3:**
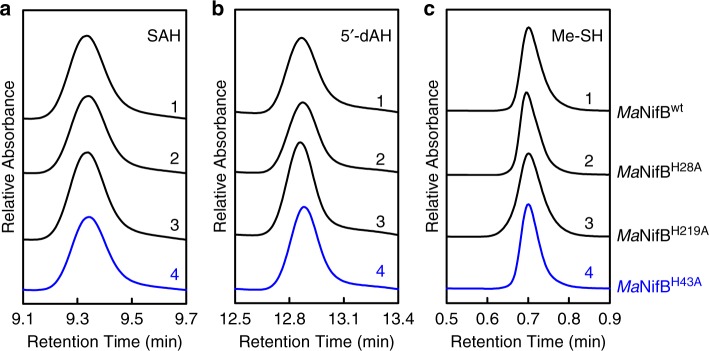
Functional properties of *Ma*NifB proteins. **a**, **b** HPLC elution profiles of SAH (**a**) and 5′-dAH (**b**) upon incubation of SAM with *Ma*NifB^wt^ (1), *Ma*NifB^H28A^ (2), *Ma*NifB^H219A^ (3), and *Ma*NifB^H43A^ (4). **c** GC analyses of methanethiol (Me-SH) formation upon acid quenching of incubation mixtures containing SAM and *Ma*NifB^wt^ (1), *Ma*NifB^H28A^ (2), *Ma*NifB^H219A^ (3), and *Ma*NifB^H43A^ (4). All products were identified using standards^13,14^. The HPLC (**a**, **b**) and GC (**c**) experiments were each performed three times independently (*n* = 3 independent samples), and representative results are shown in the figure. The protein and SAM concentrations are 0.4 mM and 4 mM, respectively, in (**a**, **b**); and 40 μM and 0.3 mM, respectively, in (**c**). See Methods for detailed compositions of these assays.

To further explore the role of His^43^ in this process, high performance liquid chromatography (HPLC) was performed to examine the products generated upon incubation of *Ma*NifB^H43A^ with SAM. Like *Ma*NifB^wt^, *Ma*NifB^H28A^, and *Ma*NifB^H219A^ (Fig. 3a, b, traces 1–3), *Ma*NifB^H43A^ (Fig. 3a, b, trace 4) can cleave SAM into *S*-adenosyl-L-homocysteine (SAH) and 5′-deoxyadenosine (5′-dAH). In addition, as observed in the cases of *Ma*NifB^wt^, *Ma*NifB^H28A^, and *Ma*NifB^H219A^ (Fig. 3c, traces 1–3), formation of methanethiol is detected upon acid quench of an incubation mixture of *Ma*NifB^H43A^ and SAM (Fig. 3c, trace 4). This observation is not particularly surprising because our previous study demonstrates that SAH, 5′-dAH and methanethiol can be generated as long as both SAM- and K2-clusters are present, even in absence of the K1-cluster. The fact that substitution of the K1-specific ligand, His^43^, with Ala does not impact the reactivities associated with the SAM- and K2-clusters is consistent with this observation and places the perturbation of the K- to L-cluster conversion by this substitution after the hydrogen atom abstraction from the K2-associated methyl group (see Supplementary Fig. 1a).

### Dual actions of histidine ligand in L-cluster maturation

**Fig. 4:**
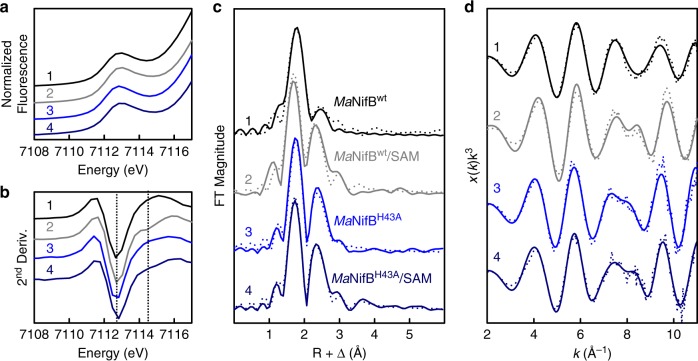
Fe K-edge XAS analysis of *Ma*NifB proteins. **a** Pre-edge regions of the normalized fluorescence spectra and (**b**) smoothed second derivatives of the pre-edge regions, (**c**) Fourier transforms of the EXAFS data (dotted) and the best fits of data (solid), and (**d**) *k*^3^-weighted EXAFS data (dotted) and the best fits of data (solid). Spectra are shown for *Ma*NifB^wt^ before (1) and after (2, designated *Ma*NifB^wt^/SAM) incubation with SAM, and *Ma*NifB^H43A^ before (3) and after (4, designated *Ma*NifB^H43A^/SAM) incubation with SAM. Note that *Ma*NifB^wt^ carries the K-cluster (a [Fe_4_S_4_] cluster pair); whereas *Ma*NifB^wt^/SAM carries the L-cluster ([Fe_8_S_9_C]). The peaks at ~7112.6 eV and ~7114.5 eV of the pre-edge regions are indicated by dashed vertical lines to illustrate the transition from a single peak at ~7112.6 eV to two peaks at ~7112.6 eV and ~7114.5 eV in the spectrum of *Ma*NifB^wt^ (**b**, 1 vs. **a**, 2), which corresponds to conversion of the K-cluster to an L-cluster upon incubation of *Ma*NifB^wt^ with SAM. Such a change is not observed in the case of *Ma*NifB^H43A^ following the same treatment with SAM (**b**, 3 vs. **a**, 4). The XAS analysis was performed three times independently (*n* = 3 independent samples), and representative results are shown in the figure. All protein samples have a concentration of 50 mg mL^−1^. All scans were taken at ~10 K. See Supporting Information for more details of EXAFS fits.

X-ray absorption spectroscopy (XAS)/extended x-ray absorption fine structure (EXAFS) analysis provided further insights into the role of His^43^ in L-cluster maturation. XAS/EXAFS analysis has proven to be a valuable tool for obtaining structural information of the cluster species related to the function and assembly of nitrogenase, and previous studies of the wildtype and variant *Ma*NifB proteins have established XAS/EXAFS parameters that can be used in combination with the EPR and biochemical data to conclusively assign cluster species and monitor cluster transformation in this protein. The x-ray absorption near edge structure (XANES) data reveal a K-edge energy for *Ma*NifB^H43A^ similar to that for *Ma*NifB^wt^ before or after incubation with SAM (Fig. 4a), suggesting a similar sulfur-rich environment in all these protein species^16^. The pre-edge feature of *Ma*NifB^H43A^ is also similar in intensity to that of *Ma*NifB^wt^ before incubation with SAM; however, its amplitude does not increase as much as that of *Ma*NifB^wt^ upon incubation with SAM (Table 1), suggesting that the transition metal center in SAM-treated *Ma*NifB^H43A^ (designated *Ma*NifB^H43A^/SAM) is less distorted away from centrosymmetry^23,24^ than that in SAM-treated *Ma*NifB^wt^ (designated *Ma*NifB^wt^/SAM). Consistent with this observation, the smoothed second derivative of the pre-edge data of *Ma*NifB^wt^/SAM transitions from a single inverted peak at ~7112.6 eV to two inverted peaks at ~7112.6 eV and ~7114.5 eV, respectively (Fig. 4b). Such a change has been attributed to the conversion of the K-cluster (with typical tetrahedral Fe-site geometries^25^) to an L-cluster (with an unusual intermediate geometry between tetrahedral and trigonal pyramidal^25^) in *Ma*NifB^wt^ upon incubation with SAM^16^. In the case of *Ma*NifB^H43A^, while a similar peak at ~7112.6 eV is observed in the second derivative before and after incubation with SAM, the line-shapes of these plots beyond 7113 eV are different than those of the corresponding *Ma*NifB^wt^ species (Fig. 4b). More importantly, the second peak at ~7114.5 eV is absent from the plot of *Ma*NifB^H43A^/SAM, although *Ma*NifB^H43A^/SAM seems to undergo a transition analogous to that of *Ma*NifB^wt^/SAM on the basis of the similar line-shapes of their second derivative plots (Fig. 4b).

**Table 1:** XANES analysis of the Fe K-edge EXAFS data.

Protein^a^	K-edge	Pre-edge area	Pre-edge peak
	eV	units	eV
*Ma*NifB^wt^	7117.9	21.3	7112.8
*Ma*NifB^wt^/SAM	7118.1	25.4	7112.9
*Ma*NifB^H43^A	7118.1	21.9	7112.8
*Ma*NifB^H43A^/SAM	7118.2	22.7	7113.0

^a^Data are fit between 7108 and 7117 eV from the experimental data. Data for *Ma*NifB^wt^ and *Ma*NifB^wt^/SAM are taken from Ref. ^16^.

Extended X-ray absorption fine structure (EXAFS) analysis of the Fe K-edges of *Ma*NifB^H43A^ provided important insights into the structural metrics of its associated cluster species. Prior to SAM treatment, *Ma*NifB^H43A^ and *Ma*NifB^wt^ display two similar features at R + Δ ~1.7 and 2.4 Å, respectively, in the Fourier transforms (FT; Fig. 4c) of their EXAFS data (Fig. 4d), although the feature of *Ma*NifB^H43A^ at R + Δ ~2.4 Å is much more prominent than that of *Ma*NifB^wt^. For *Ma*NifB^H43A^, these FT features can be best fit with Fe–S and Fe∙∙∙Fe scatterers at 2.29 and 2.71 Å, respectively; whereas for *Ma*NifB^wt^, they are best fit with Fe–S scatterers at 2.29 Å and Fe∙∙∙Fe scatterers at 2.51 and 2.69 Å, respectively (Table 2). Upon incubation with SAM, *Ma*NifB^H43A^/SAM and *Ma*NifB^wt^/SAM seemingly undergo similar changes, both showing extra FT features at R + Δ ~3.0 and ~3.5 Å; yet, while *Ma*NifB^wt^/SAM displays a substantially increased intensity and a clear shift of its FT feature at R + Δ ~2.4 Å, the corresponding FT feature of *Ma*NifB^H43A^/SAM remains largely unchanged (Fig. 4c). The differences between the two SAM-treated *Ma*NifB species are clearly illustrated in the best fits of their EXAFS data: *Ma*NifB^H43A^/SAM is best modeled with two types of Fe–S scatterers at 2.27 and 3.88 Å, respectively, and one type of Fe∙∙∙Fe scatterers at 2.69 Å; whereas *Ma*NifB^wt^/SAM is best modeled with one type of Fe–S scatterers at 2.23 Å and two types of Fe∙∙∙Fe scatterers at 2.64 and 3.70 Å, respectively (Table 2). Most notably, the long-range Fe∙∙∙Fe distance at 3.70 Å, which originates from the intercubane scattering between the six carbide-coordinated Fe atoms at the cofactor core^25^, is present only in *Ma*NifB^wt^/SAM but absent from *Ma*NifB^H43A^/SAM. This observation suggests that unlike *Ma*NifB^wt^, *Ma*NifB^H43A^ does not enable the formation of an L-cluster with a μ_6_-coordinated central carbide in place upon incubation with SAM. In support of this argument, *Ma*NifB^H43A^ carries clusters with short-range Fe∙∙∙Fe distances that are characteristic of the [Fe_4_S_4_] clusters before and after incubation with SAM (Table 2). However, modeling of the cluster species on *Ma*NifB^H43A^/SAM, contrary to that of the cluster species on *Ma*NifB^H43A^, requires the inclusion of an extra Fe–S distance at 3.88 Å (Table 2) that corresponds to the distance from a sulfide to a Fe at the opposite vertex of a [Fe_4_S_4_] cluster. The appearance of such a distance is consistent with an increased order of the two K-cluster units (i.e., K1 and K2), or a further processing of these [Fe_4_S_4_] units into a cluster intermediate between the K- and L-clusters on *Ma*NifB^H43A^/SAM; more importantly, it highlights an overall homogeneity of the cluster species on *Ma*NifB^H43A^/SAM, as this Fe–S distance can only be observed when the clusters are well-ordered and, therefore, uniform in nature. Overall, the mean squared deviations (σ^2^) of the S and Fe scatterers are rather small (<5 × 10^−3^ Å^2^; see Supplementary Tables 1 and 2), which further supports the homogeneity of the cluster species in the *Ma*NifB proteins. Three most likely configurations can be proposed for this cluster intermediate: (i) a partial [Fe_4_S_3_] cluster pair bridged by two sulfide (S^2-^) atoms and a carbon (CH_x_) species, (ii) a face-on [Fe_4_S_4_] cluster pair bridged by a CH_*x*_ species, and (iii) a vertex-on [Fe_4_S_4_] cluster pair bridged by a CH_x_ species, all of which lack the characteristic cofactor core structure that is defined by an interstitial carbide coordinated with six Fe atoms (Supplementary Fig. 4). Given the homogeneity of the cluster species on *Ma*NifB^H43A^/SAM, it is likely that one of these proposed models will be identified through future structural characterization of this protein.

**Table 2:** Best fits of the Fe K-edge EXAFS data.

Protein^a^	Fe–S			Fe•••Fe			Fe•••Fe		
	N	R(Å)	σ^2^ (10^−3^)	N	R(Å)	σ^2^ (10^−3^)	N	R(Å)	σ^2^ (10^−3^)
*Ma*NifB^wt^	3.8	2.29	8.19	1	2.51	5.82	1.5	2.69	4.35
*Ma*NifB^wt^/SAM	3.1	2.23	4.23	3.5	2.64	7.87	1.5	3.70	7.89
M*a*NifB^H43A^	3	2.29	2.98	2	2.71	4.02	–	–	–
*Ma*NifB^H43A^/SAM	3	2.27	4.18	2	2.69	4.19	–	–	–
	1	3.88	1.53	–	–	–	–	–	–

^a^Data is fit between *k* = 2–11.2 Å^−1^. Data for *Ma*NifB^wt^ and *Ma*NifB^wt^/SAM are taken from Ref. ^16^. See Supplementary Tables 1 and 2 for details of EXAFS data fits for *Ma*NifB^H43A^ and *Ma*NifB^H43A^/SAM, respectively.

## Discussion

Formation of a cluster intermediate on the SAM-treated *Ma*NifB^H43A^ that is distinct from both the K- and L-clusters implies that His^43^ serves as a key structural element and/or reaction component during the process of cofactor core formation on *Ma*NifB. The substantially increased intensity of the FT feature of *Ma*NifB^H43A^ at R + Δ ~2.4 Å relative to that in *Ma*NifB^wt^ prior to incubation with SAM (see Fig. 4c) points to a much stronger Fe∙∙∙Fe scattering that results from a much closer distance between the two [Fe_4_S_4_] units of the K-cluster in *Ma*NifB^H43A^ than those in *Ma*NifB^wt^. Such a change in the distance and/or orientation of the K1-cluster relative to that of the K2-cluster apparently presents a challenge for *Ma*NifB^H43A^ to initiate a proper coupling between the two K-cluster units. The His^43^ ligand, therefore, could play a steric role in keeping the two K-cluster units in the correct distance/orientation to each other by either indirectly pulling the K1-cluster away from the K2-cluster via its ligand capacity or directly separating the K1- and K2-clusters with its bulky imidazole ring (Supplementary Fig. 4, ➀). Additionally, given our observation of a loss of the nitrogen ligand upon conversion of the K-cluster to an L-cluster on *Ma*NifB^11^, His^43^ may lose its coordination to the K1-cluster via protonation, thereby freeing up K1 for the subsequent coupling with K2 into an L-cluster, and facilitating the release of the completed L-cluster to the next biosynthetic apparatus for further maturation. As such, His^43^ likely functions as a molecular switch via reversible protonation and deprotonation events, securing the cluster in place in its deprotonated state while giving the cluster certain structural flexibility in its protonated state to accommodate the different states required for cluster conversion. A similarly labile nitrogen ligand is also found in mitoNEET, where protonation of a His ligand to a [Fe_2_S_2_] cluster permits transfer of the cluster to downstream acceptor proteins^26,27^.

In light of this proposal, it is interesting to consider a coupling of the function of His^43^ as a molecular switch with another role of this residue in cofactor core formation, one that is involved in the further deprotonation/dehydrogenation of the initial methylene radical to yield the interstitial carbide at the center of the L-cluster. The observation that the two K-cluster units in SAM-treated *Ma*NifB^H43A^ become more aligned with each other but remain largely separate FeS cubanes in character (Supplementary Fig. 4, ➁) is consistent with an interruption of the carbide formation/insertion process that is required for the coupling/rearrangement of K1 and K2 into the geometry of a cofactor core. Consequently, the cluster is rendered in an unfinished state with the carbon intermediate (CH_*x*_) not fully deprotonated or dehydrogenated and attached to one or both of the K-cluster units (Supplementary Fig. 4, ➂). It is important to note that, other than His^43^, additional residues may also be involved in processing the carbon intermediate to an interstitial carbide, as substitution of His^28^ with Ala apparently reduces the efficiency of L-cluster formation on NifB by 41% (see Fig. 2b). A proton relay mechanism involving multiple histidine (or equivalent) residues may be employed in this case to facilitate efficient deprotonation/dehydrogenation of the initial methylene radical to eventually yield a carbide in the center of the L-cluster. While the role of His^43^ and other relevant players in the cofactor core formation process is yet to be elucidated, the results of this study provide an important framework for further investigations into the unique radical chemistry underlying the formation of the core structure of the nitrogenase cofactor. Knowledge obtained from these studies will contribute to a better understanding of the mechanism of nitrogenase and shed important light on the mechanisms of analogous biological systems.

## Methods

### General information

Unless otherwise specified, all chemicals were purchased from Sigma-Aldrich (St. Louis, MO) and Thermo Fisher Scientific (Waltham, MA), and all experiments were performed under an Ar atmosphere using Schlenk techniques and a glove box operating at <3 ppm O_2_.

### Cell growth and protein purification

*E. coli* strains expressing His-tagged *Ma*NifB^wt^ (strain YM114EE), *Ma*NifB^H28A^ (strain YM242EE), *Ma*NifB^H43A^ (strain YM244EE), *Ma*NifB^H219A^ (strain YM246EE), *Ma*NifB^K1^ (strain YM165EE) and *Ma*NifB^K1-H43A^ (strain YM307EE) were grown in 10-L batches in Difco LB medium containing 100 mg L^-1^ ampicillin (BD Biosciences) in a BIOFLO 415 fermenter (New Brunswick Scientific) at 37 °C, with 200 rpm agitation and 10 L˄min^-1^ airflow. When OD_600_ reached 0.5, the temperature was lowered to 25 °C before expression of the wildtype and variant *Ma*NifB proteins was induced by addition of 25 µM IPTG. Expression of proteins was allowed to continue for 16 h before cells were harvested by centrifugation using a Thermo Fisher Scientific Legend XTR centrifuge. Subsequently, His-tagged *Ma*NifB proteins were purified by immobilized metal affinity chromatography (IMAC)^9,10^.

### SDS-PAGE analysis

The purified wildtype and variant *Ma*NifB proteins were subjected to sodium dodecyl sulfate-polyacrylamide gel electrophoresis (SDS-PAGE) analysis on a 4-20% precast Tris-glycine gel (Bio-Rad).

### Iron determination

The iron contents of the FeS-reconstituted wildtype and variant *Ma*NifB proteins were determined by inductively coupled plasma optical emission spectroscopy (ICP-OES) using a Thermo Scientific iCAP7000. Stock solutions of elemental iron (1 mg mL^−1^, Inorganic Ventures) were diluted to make standard solutions for calibration. Each protein sample was mixed with 100 µL concentrated sulfuric acid (H_2_SO_4_) and 100 µL concentrated nitric acid (HNO_3_) and heated at 250 °C for 30 min. This procedure was repeated until the solutions became colorless. The solution was then cooled to room temperature and diluted to a total volume of 10 mL with 2% HNO_3_ prior to sample analysis.

### FeS cluster reconstitution

The purified wildtype or variant *Ma*NifB was treated with 20 mM bathophenanthroline disulfonate, an iron chelator, in a buffer containing 2 mM dithionite (DT; Na_2_S_2_O_4_), 50 mM Tris-HCl (pH 8.0) and 500 mM NaCl, followed by incubation at room temperature for 1 h to remove the endogenous FeS clusters associated with the protein. Subsequently, this mixture was diluted with a buffer containing 50 mM Tris-HCl (pH 8.0) and loaded on a Q Sepharose column (GE Healthcare). The column was then washed with a buffer containing 2 mM DT, 50 mM Tris-HCl (pH 8.0) and 50 mM NaCl prior to elution of the *Ma*NifB protein with a buffer containing 50 mM Tris-HCl (pH 8.0) and 500 mM NaCl. Dithionite was removed by running protein through a Sephadex G-25 (GE Healthcare) column equilibrated with 50 mM Tris-HCl (pH 8.0) and 10% glycerol. Reconstitution of the wildtype or variant *Ma*NifB protein with synthetic [Fe_4_S_4_] clusters^15^ was carried out by adding a dimethylformamide (DMF) solution of synthetic [Fe_4_S_4_] cluster dropwise at a molar ratio of 5:1 to the *Ma*NifB protein in a buffer containing 20 mM β-mercaptoethanol and 50 mM Tris-HCl (pH 8.0), with continuous stirring on ice. After incubation on ice for 1 h, the reaction mixture was diluted with a buffer containing 2 mM DT and 50 mM Tris-HCl (pH 8.0) and loaded on a Q Sepharose column. The column was then washed with a buffer containing 2 mM DT, 50 mM Tris-HCl and 50 mM NaCl prior to elution of the reconstituted *Ma*NifB with a buffer containing 2 mM DT, 50 mM Tris-HCl (pH 8.0) and 500 mM NaCl. Reconstituted wildtype and variant *Ma*NifB proteins were subjected to metal determination, activity assays and EPR analysis.

### Cofactor maturation assays

The cofactor maturation assay contained, in a total volume of 1.0 mL, 25 mM Tris-HCl (pH 8.0), 20 mM DT, 3.5 mg FeS-reconstituted wildtype or variant *Ma*NifB, 10 mM SAM, 2 mg Δ*nifB Av*NifEN, 1.4 mg NifH, 0.8 mM ATP, 1.6 mM MgCl_2_, 10 mM creatine phosphate, 8 units creatine phosphokinase, 0.3 mM homocitrate, 0.3 mM Na_2_MoO_4_ and 0.5 mg Δ*nifB Av*NifDK. This mixture was incubated at 30 °C for 30 min before it was examined for enzymatic activities^9,10^.

### *S*-adenosyl-L-methionine (SAM) cleavage assays

The SAM cleavage assay contained, in a total volume of 0.3 mL, 25 mM Tris-HCl (pH 8.0), 5% glycerol (v/v), 40 µM wildtype or variant *Ma*NifB, and 0.3 mM SAM. Assays were incubated at 25 °C for 60 min with intermittent mixing, before they were terminated by filtration through Amicon Ultra 30,000 MWCO centrifugal filters. Samples were then supplemented by trifluoroacetic acid (TFA) to a concentration of 0.14% before being analyzed by a Thermo Scientific Dionex Ultimate 3000 UHPLC system equipped with an Acclaim 120 C18 column (4.6 × 100 mm, 5-µm particle size). The flow rate of buffer was 0.5 mL˄min^-1^, and the column was kept at 30 °C. The column was equilibrated with 98% buffer A (50 mM KH_2_PO_4_, pH 6.6) and 2% buffer B (100% methanol) before each injection of a 100-µL sample. After sample injection, a linear gradient of 2–60% buffer B was applied over 20 min, followed by 8 min of isocratic flow with 60% buffer B and a linear gradient of 60–2% buffer B over 4 min. Elution of products was monitored at a UV wavelength of 254 nm. After each run, the column was equilibrated for 5 min with 2% buffer B before the injection of the next sample.

### Acid quench experiments

Detection of *Ma*NifB-dependent production of methanethiol was performed^14^. First, excess DT was removed from wildtype and variant *Ma*NifB via gel filtration with Sephadex G-25 fine resin that was equilibrated with a buffer containing 25 mM Tris-HCl (pH 8.0). Immediately following the removal of excess reductant, 40 nmol of *Ma*NifB was added to a sealed 300-μL glass vial that contained 400 nmol SAM in a total volume of 100 μL. These 100-μL reactions were then incubated for 30 min at 25 °C before being quenched by 25 μL of 1 M HCl. To observe the formation of the volatile methanethiol, the acid-quenched samples were incubated at 60 °C for 15 min and equilibrated to room temperature for 10 min before the entire headspace was injected by a gas-tight syringe onto a GC–MS (Thermo-Fisher Scientific Trace 1300 GC connected to a Thermo-Fisher Scientific ISQ QD single quadrupole mass spectrometry) with a Restek Rxi-1ms column (30 m, 0.32 mm ID, 4.0 μm df). The GC inlet and oven temperatures were maintained at 30 °C, while the mass spectrometry transfer line and ion source were maintained at 250 °C. Total ion chromatograms were generated under SIM conditions in electron ionization mode, and methanethiol was detected at an *m/z* ratio of 47. The base peaks were selected on the basis of the characterization of standard samples (Sigma-Aldrich) under full scan conditions and comparison to those reported in the National Institute of Standards and Technology database.

### Electron paramagnetic resonance (EPR) analysis

Sample preparation was carried out in a Vacuum Atmospheres glove box with less than 1 ppm O_2_ and flash frozen in liquid nitrogen before analysis. The dithionite-reduced samples were prepared by incubating 50 μM wildtype or variant *Ma*NifB with 40 mM SAM for 15 min, followed by re-isolation of *Ma*NifB into a buffer containing 50 mM Tris-HCl (pH 8.0), 500 mM NaCl, and 2 mM dithionite (DT). The indigo disulfonate (IDS)-oxidized samples were prepared by incubating the re-isolated *Ma*NifB with excess IDS for 5 min, followed by removal of excess IDS was using a Sephadex G-25 desalting column. The DT-reduced or IDS-oxidized *Ma*NifB sample was then concentrated to 15 mg mL^−1^, followed by transfer of the sample into EPR tubes. The samples were then flash frozen and stored in liquid nitrogen. CW EPR spectra were recorded by an ESP 300 E_z_ spectrophotometer (Bruker) interfaced with an ESR-9002 liquid-helium continuous-flow cryostat (Oxford Instruments) using a microwave power of 5 mW (IDS-oxidized samples) or 50 mW (IDT-reduced samples), a gain of 5 × 10^4^, a modulation frequency of 100 kHz, and a modulation amplitude of 5 G. Five scans was recorded for each sample in perpendicular mode at 20 K (IDS-oxidized samples) or 10 K (DT-reduced samples) using a microwave frequency of 9.62 GHz.

All pulse EPR studies were carried out at the UC Davis CalEPR center, using a Bruker EleXsys E580 pulse EPR spectrometer equipped with an Oxford-CF935 liquid-helium cryostat and an ITC-503 temperature controller. Pulse data were collected using a Bruker MS5 probe (X-band) or an R.A. Isaacson-designed cylindrical TE011 resonator (Q-band)^28^ adapted for pulse EPR in an Oxford Instruments CF935 cryostat. Three-pulse ESEEM spectra were collected using the pulse sequence π/2-τ-π/2-T-π/2-τ-echo where the delay time, T, was increased by 16 ns steps. ESEEM spectra were recorded at 10 K, τ = 128-144 ns (values chosen to minimize proton modulations to the spectra), π/2 = 12 ns, and a microwave frequency of 9.3366 GHz. Spectral processing (subtraction of a background exponential, application of a hamming window, and FFT) were performed using the EasySpin 5.2.27 toolbox in Matlab R2019a^29^.

### X-ray absorption spectroscopy (XAS) analysis

The XAS samples were prepared the same way as the EPR samples (see above). The sample concentrations were 50 mg mL^−1^. Fe K-edge X-ray absorption spectra were collected on SSRL beam line 7-3 using a 30-element solid state Ge detector (Canberra) with a SPEAR3 storage ring current of ~500 mA at an energy of 3.0 GeV. The BL7-3 optics consists of a flat, bent, harmonic rejection vertically collimating Rh-coated Si M_0_ mirror and a liquid nitrogen cooled double crystal Si(220) monochromator. A total of 7 and 6 scans, respectively, were collected for *Ma*NifB^H43A^ before and after incubation with SAM (designated *Ma*NifB^H43A^ and *Ma*NifB^H43A^/SAM). All scans were taken between 6882 and 8000 eV at ~10 K using an Oxford Instruments CF1208 continuous flow liquid-helium cryostat using a closed-cycle cooled He gas loop. An iron foil was placed in the beam pathway prior to the ionization chamber I_0_ and scanned concomitantly for an energy calibration, with the first inflection point of the edge assigned to 7112.0 eV. A Soller slit with a 3 μm Mn filter was used to increase the signal-to-noise ratio of the spectra. Photoreduction was monitored by scanning the same spot on the sample twice and comparing the first derivative peaks associated with the edge energy during data collection.

The detector channels from the scans were examined, calibrated and averaged using EXAFSPAK^30^ and then processed for EXAFS analysis using PYSPLINE^31^ to extract χ(*k*). PYSPLINE was used to subtract a second-order background from the entire range of data and subsequently generate a spline function to model background absorption through the EXAFS region. A four-region spline was chosen with 2, 3, 3 order polynomials over the post edge region, and the data were normalized to have an edge jump of 1.0 at 7130 eV. Following a modified data analysis protocol^25^, the Fe K-edge EXAFS data for the clusters associated with *Ma*NifB^H43A^ and *Ma*NifB^H43A^/SAM were generated by subtracting the *k*-weighted EXAFS data, χ(*k*), of *Ma*NifB^SAM^ (an *Ma*NifB variant carrying only the SAM-cluster^11^) from the χ(*k*) of the samples in a 1:2 ratio on the basis of the proportionate iron quantity for each cluster species (i.e., 4 Fe for the SAM-cluster and 8 Fe for the K-clusters). Theoretical phase and amplitude parameters for a given absorber–scatterer pair were calculated using FEFF 8.40^32^ and subsequently applied to the nonlinear least squares Opt fitting program of the EXAFSPAK package during curve fitting. Parameters for each species were calculated using an appropriate model derived from either the crystal structure of the M-cluster in NifDK (PDB code 3U7Q)^5^, where the Mo atom was exchanged for an Fe atom, or from the [Fe_4_S_4_] cluster in NifH (PDB code 1G5P)^33^ because there are no available crystal structures of *Ma*NifB. In all analyses, the coordination number of a given shell (*N*) was a fixed parameter and was varied iteratively in integer steps, whereas the bond lengths (*R*) and mean-square deviation (σ^2^) were allowed to freely float. The estimated uncertainties in *R*, σ^2^, and *N* are 0.02 Å, 0.1 × 10^-3^ Å^2^, and 20%, respectively. The amplitude reduction factor S_0_ was fixed at 1.0 for the Fe K-edge data, whereas the edge-shift parameter Δ*E*_0_ was allowed to float as a single value for all shells. Thus, in any given fit, the number of floating parameters was typically equal to 2 × number of shells + 1. The goodness of fit (GOF) parameters were calculated as follows:

1 $$ F = \sqrt {\sum k^6\left( {\chi _{{\rm{exp}}} - \chi _{{\rm{calc}}}}\right)^2},$$

2 $$F^\prime = \sqrt {\sum k^6\left(\chi _{{\rm{exp}}} - \chi _{{\rm{calc}}}\right)^2/\sum k^6\left(\chi _{{\rm{exp}}}\right)^2}.$$

The Fe K-edge data were analyzed with a *k* range of 2–11.2 Å^−1^ (ΔR = 0.17 Å) to allow comparison between previously reported data^16^, although the data could be analyzed with higher resolution with a *k* range of 2–14 Å^−1^ for *Ma*NifB^H43A^ and *Ma*NifB^H43A^/SAM. Pre-edge analysis was performed on the Fe K-edge fluorescence data normalized to have an edge jump of 1.0 at 7130 eV in PYSPLINE. The pre-edge features were fit as described elsewhere^23^ between 7108 and 7117 eV using the Fityk^34^ program with pseudo-Voigt functions composed of 50:50 Gaussian/Lorentzian functions.

### Reporting summary

Further information on research design is available in the Nature Research Reporting Summary linked to this article.

## Supplementary information

Supplementary Information

Reporting Summary

## Data Availability

The authors declare that all data supporting the findings of this study are available within the article and its Supplementary Information files and from the corresponding authors upon reasonable request. The National Institute of Standards and Technology database is available at https://www.nist.gov/data. Parameters used for cluster modeling are available at https://www.rcsb.org/ using PDB IDs 3U7Q and 1G5P.
