# Supplementary material for: Identity and function of an essential nitrogen ligand of the nitrogenase cofactor biosynthesis protein NifB
Source: Nat Commun. 2020 Apr 9;11:1757. doi: 10.1038/s41467-020-15627-9 (PMC7145814; doi:10.1038/s41467-020-15627-9)
Supplement: Supplementary file 1 — Supplementary Information [file 41467_2020_15627_MOESM1_ESM.pdf]

SUPPLEMENTARY INFORMATION

Identity and function of an essential nitrogen ligand of the nitrogenase cofactor biosynthesis protein NifB

Lee A. Rettberg,[1,a] Jarett Wilcoxen,[2,a,b] Andrew J. Jasniewski,[1,a] Chi Chung Lee,[1] Kazuki Tanifuji,[1] Yilin Hu,[1,*] R. David Britt,[2,*] Markus W. Ribbe[1,3*]

[1]Department of Molecular Biology and Biochemistry, University of California, Irvine, CA 92697-3900; [2]Department of Chemistry, University of California, Davis, CA 95695; [3]Department of Chemistry, University of California, Irvine, CA 92697-2025

[a]These authors contributed equally.

[b]Present address: Department of Chemistry and Biochemistry, University of Wisconsin, Milwaukee, WI 53211

*Correspondence should be sent to rdbritt@ucdavis.edu, yilinh@uci.edu or mribbe@uci.edu.

SUPPLEMENTARY FIGURES

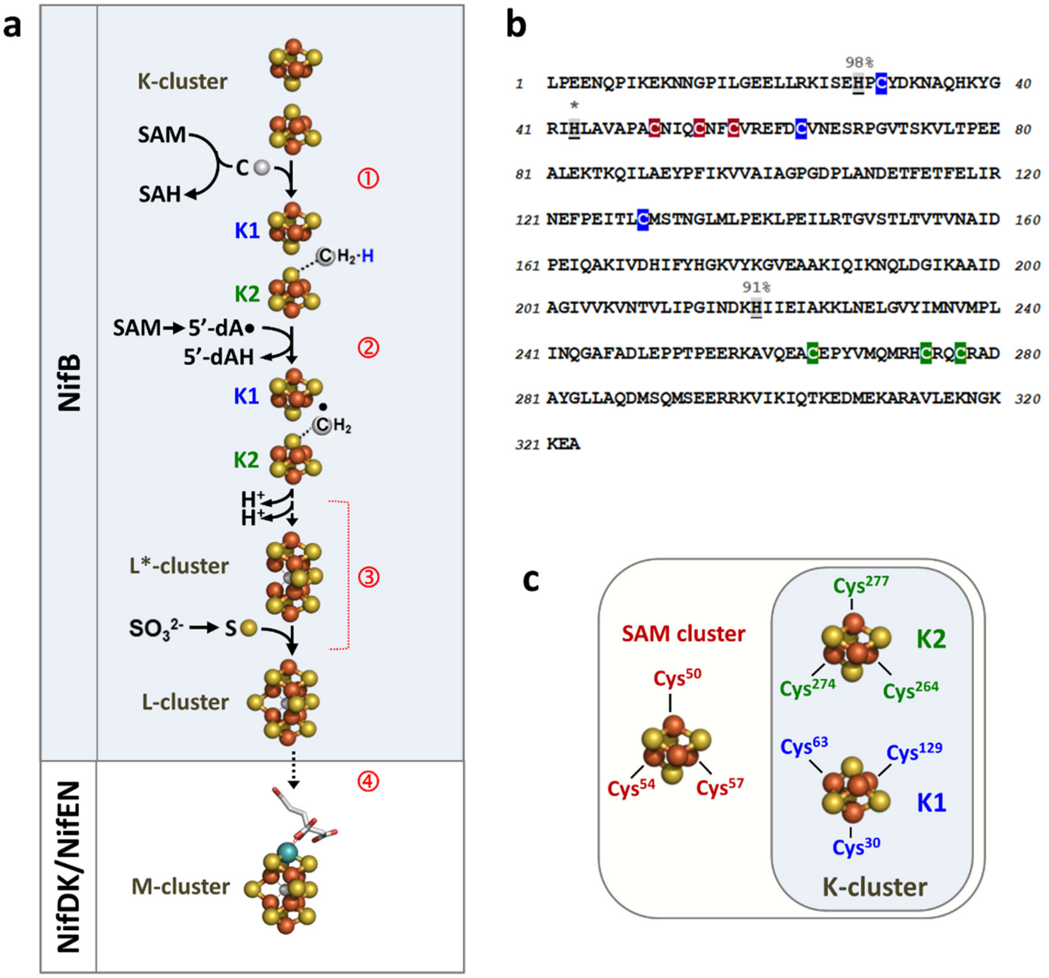

Supplementary Figure 1 | (**a**) Proposed mechanism of nitrogenase M-cluster biosynthesis. On NifB, the coupling of the two $[Fe_4S_4]$ modules the K-cluster (designated K1- and K2-cluster, respectively) into an 8Fe L*-cluster ($[Fe_8S_8C]$) occurs concomitant with the insertion of an interstitial carbide. Insertion of carbide begins with transfer of a methyl group from SAM to the K2-cluster (①) and continues with hydrogen atom abstraction from the K2-associated methyl group by a SAM-derived 5′-dA• radical (②). Further deprotonation/dehydrogenation of the carbon radical gives rise to a carbide in the center of the L*-cluster concomitant with insertion of a sulfite (SO_3^{2-}) -derived '9th sulfur' into the belt region of this cluster, leading to the formation of an 8Fe L-cluster ($[Fe_8S_9C]$) on NifB (③). The L-cluster is then transferred to NifEN and further matured into an M-cluster ($[MoFe_7S_9C]$) upon insertion of Mo and homocitrate prior to transfer of the M-cluster from NifEN to its target binding site in NifDK (④). Fe, orange; Mo, cyan; S, yellow; C, grey; N, blue; O, red. (**b**) The primary sequence of *Ma*NifB, with the Cys ligands for the SAM-, K1- and K2-cluster highlighted in red, blue and green, respectively[11]. The His residues that are conserved among 45 NifB sequences from various organisms[11] are highlighted in grey, with the 100% conserved residues indicated with a *, and others noted for the percentages of conservation among these organisms. (**c**) Schematic presentations of the SAM-, K1-, and K2-modules in *Ma*NifB. A 3-Cys ligation pattern was identified for all three modules[11]. The ligands for each module are color-coded as depicted in b.

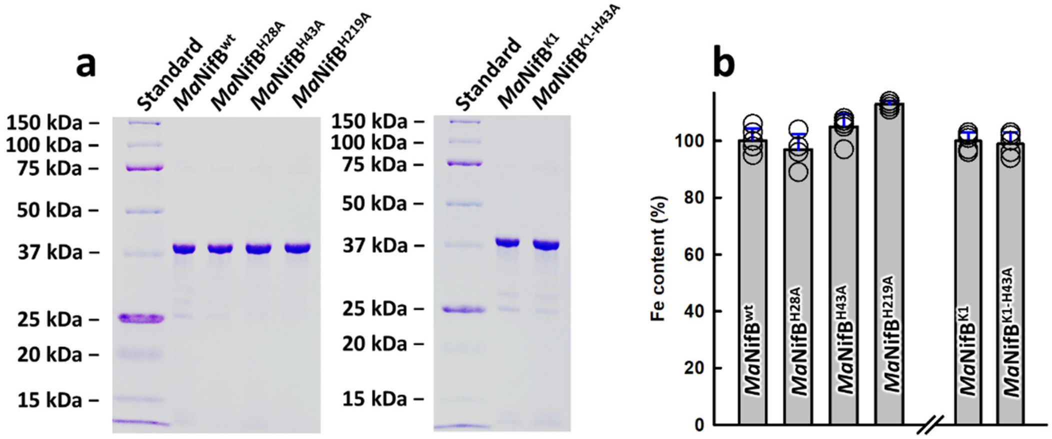

Supplementary Figure 2 | SDS PAGE (**a**) and Fe content (**b**) of the wildtype and variant *Ma*NifB proteins. The Fe contents of *Ma*NifB^{wt} (11.9 + 1.1 mol Fe/mol protein) and *Ma*NifB^{K1} (3.9 + 0.1 mol Fe/mol protein) are set to 100% and compared with the Fe contents of their respective variant forms (b). The SDS PAGE analysis was performed three times independently (n=3 independent samples), and representative results are shown (a). The metal analysis was performed five times independently (n=5 independent samples), and data are shown as mean + S.D. (b). A total of 2 µg of each protein was loaded onto the SDS polyacrylamide gel (a), and a total of 2 mg of each protein was used for metal analysis (b).

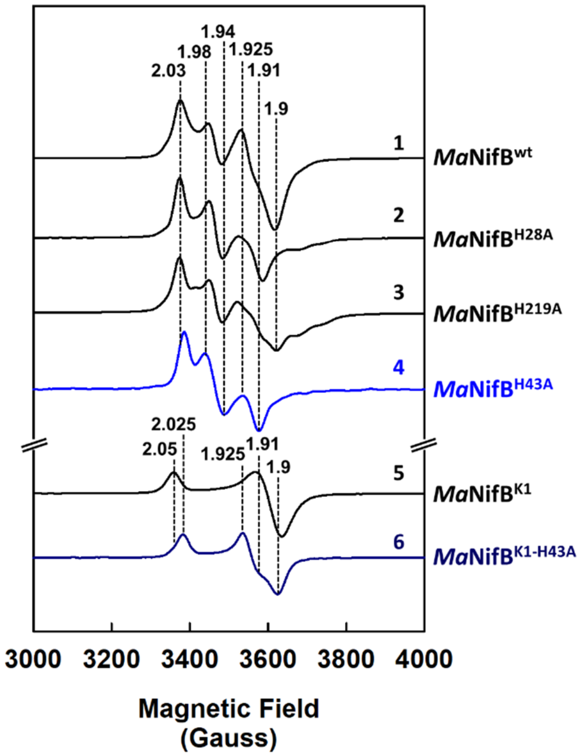

Supplementary Figure 3 | EPR spectra of dithionite-reduced *Ma*NifB proteins. Shown are the CW EPR spectra of *Ma*NifB^{wt} (1), *Ma*NifB^{H28A} (2), *Ma*NifB^{H219A} (3), *Ma*NifB^{H43A} (4), *Ma*NifB^{K1} (5) and *Ma*NifB$^{K1\text{-}H43A}$ (6) in the dithionite-reduced state. The EPR analysis was performed three times independently (n=3 independent samples), and representative results are shown. All protein samples have a concentration of 15 mg^mL^{-1}. The spectra were recorded at 50 mW and 10 K. The *g* values are indicated (dashed vertical lines).

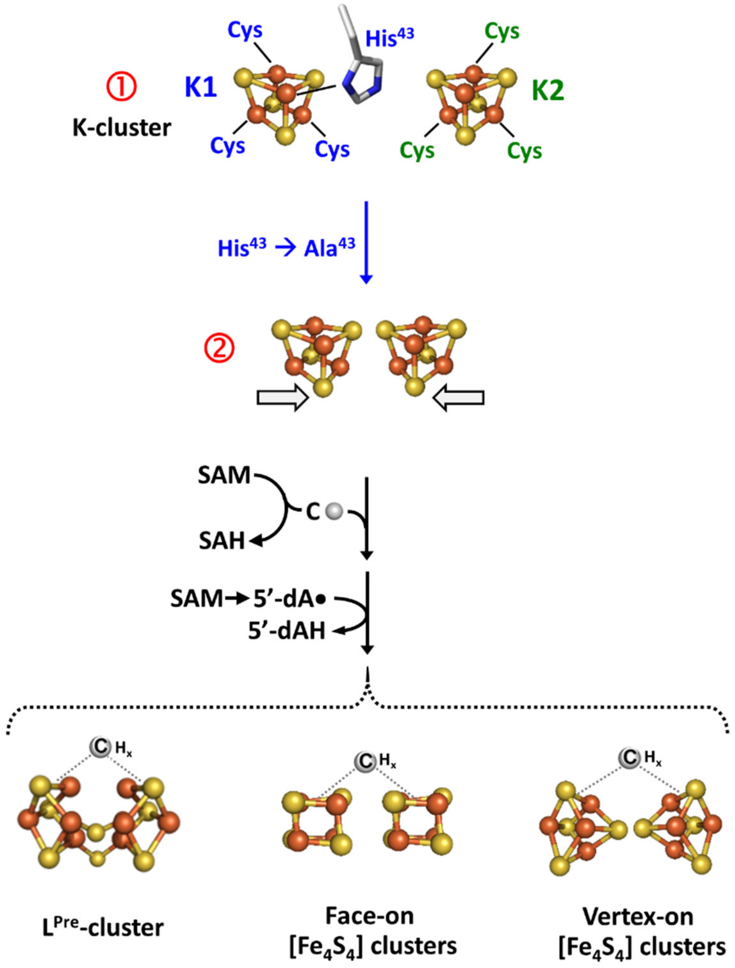

Supplementary Figure 4 | Proposed effect of the substitution of His^{43} with Ala in *Ma*NifB on cluster conversion. On *Ma*NifB^{wt}, the K1- and K2 clusters are kept in proper distance and/or orientation to each other by the His^{43} ligand of K1 either through its ligand capacity or via its bulky imidazole ring (①). The replacement of the His^{43} ligand with Ala renders the K1- and K2-clusters in a closer proximity and/or an incorrect orientation to each other, as suggested by our XAS/EXAFS analysis of *Ma*NifB^{H43A} prior to treatment with SAM (②). Incubation of *Ma*NifB^{H43A} with SAM results in a conformational rearrangement of the two K-cluster units (*i.e.*, K1 and K2) into a cluster intermediate that represents a precursor to the [Fe_8S_9C] L-cluster (③). Shown are three plausible models of the cluster intermediate with configurations consistent with our XAS/EXAFS analysis of the SAM-treated *Ma*NifB^{H43A}: (*left*) a so-called L^{Pre}-cluster that has an analogous topology to the L-cluster but does not have the μ_6-coordinated interstitial carbide and the '9th sulfur' (one of the belt sulfurs) in place; (*middle*) a face-on pair of [Fe_4S_4] clusters; and (*right*) a vertex-on pair of [Fe_4S_4] clusters. The observation of SAH and 5'-dAH as the products of SAM cleavage by *Ma*NifB^{H43A} (*see* Figure 3a, b), along with that of Me-SH formation upon acid quenching of the SAM-treated *Ma*NifB^{H43A} (*see* Figure 3c), points to attachment of a partially dehydrogenated/deprotonated carbon intermediate (CH_x) to the Fe and/or S atom of the K1- and/or K2-cluster (gray dotted lines).

SUPPLEMENTARY TABLES

Supplementary Table 1 │ Fit parameters for the EXAFS data of *Ma*NifB^{H43A} between *k*=2–11.2 Å^{-1}.

	Fe–S			Fe•••Fe				GOF	
Fit	N	R(Å)	$\sigma^2(10^{-3})$	N	R(Å)	$\sigma^2(10^{-3})$	ΔE_0	F	F'
1	1	2.24	-3.10				-15.8	1341	629
2	2	2.25	0.49				-15.0	1144	581
3	3	2.25	3.04				-14.4	1120	575
4	4	2.25	5.18				-13.9	1173	588
5	2	2.28	0.76	1	2.71	0.78	-8.06	443	362
6	3	2.29	3.47	1	2.72	0	-7.20	305	300
7[a]	3	2.29	2.98	2	2.71	4.02	-7.26	206	247
8	4	2.29	5.25	2	2.72	3.48	-7.05	221	255
9	4	2.29	4.93	3	2.72	6.29	-6.95	237	265
10	3	2.28	2.79	3	2.71	6.79	-7.30	184	232

[a]Fit 7 gives the most reasonable fit of the experimental data.

Supplementary Table 2 │ Fit parameters for the EXAFS data of *Ma*NifB^{H43A}/SAM between *k*=2–11.2 Å^{-1}.

	Fe–S			Fe•••Fe				GOF	
Fit	N	R(Å)	$\sigma^2(10^{-3})$	N	R(Å)	$\sigma^2(10^{-3})$	ΔE_0	F	F'
1	1	2.23	-2.30				-17.2	947	626
2	2	2.23	1.59				-16.4	888	606
3	3	2.23	4.47				-15.7	930	620
4	4	2.24	7.05				-14.6	1009	646
5	2	2.27	1.80	1	2.69	0.65	-8.80	227	306
6	3	2.27	4.78	1	2.70	0.02	-8.55	184	276
7	3	2.27	4.23	2	2.69	4.29	-8.82	174	268
8	3	2.26	3.98	3	2.69	7.25	-8.78	210	294
9[a]	3	2.27	4.18	2	2.69	4.19	-8.73	152	250
	1	3.88	1.53						
10	3	2.27	4.15	2	2.69	4.25	-8.90	160	257
	2	3.89	6.78						
11	3	2.26	3.93	3	2.69	7.14	-9.04	191	280
	1	3.88	1.96						
12	4	2.27	6.61	2	2.70	3.81	-8.44	225	305
	1	3.89	1.11						

[a]Fit 9 gives the most reasonable fit of the experimental data.
